# Investigating the relationship between climate, stand age, and temporal trends in masting behavior of European forest trees

**DOI:** 10.1111/gcb.14945

**Published:** 2020-01-17

**Authors:** Mario B. Pesendorfer, Michał Bogdziewicz, Jakub Szymkowiak, Zbigniew Borowski, Władysław Kantorowicz, Josep M. Espelta, Marcos Fernández‐Martínez

**Affiliations:** ^1^ Institute of Forest Ecology Department of Forest and Soil Sciences University of Natural Resources and Life Sciences Vienna Austria; ^2^ Cornell Lab of Ornithology Ithaca NY USA; ^3^ Smithsonian Migratory Bird Center National Zoological Park Washington DC USA; ^4^ Department of Systematic Zoology Adam Mickiewicz University Poznań Poland; ^5^ Population Ecology Lab Faculty of Biology Adam Mickiewicz University Poznań Poland; ^6^ Department of Forest Ecology Forest Research Institute Raszyn Poland; ^7^ Department of Silviculture and Genetics of Forest Trees Forest Research Institute Raszyn Poland; ^8^ CREAF Cerdanyola del Vallès Catalonia Spain; ^9^ PLECO (Plants and Ecosystems) Department of Biology University of Antwerp Wilrijk Belgium

**Keywords:** aging, climate change, demography, drought, mast‐seeding, seed production, temperate forests

## Abstract

Masting—temporally variable seed production with high spatial synchrony—is a pervasive strategy in wind‐pollinated trees that is hypothesized to be vulnerable to climate change due to its correlation with variability in abiotic conditions. Recent work suggests that aging may also have strong effects on seed production patterns of trees, but this potential confounding factor has not been considered in previous times series analysis of climate change effects. Using a 54 year dataset for seven dominant species in 17 forests across Poland, we used the proportion of seed‐producing trees (PST) to contrast the predictions of the climate change and aging hypotheses in *Abies alba*,* Fagus sylvatica*,* Larix decidua*,* Picea abies*,* Pinus sylvestris*,* Quercus petraea*, and *Quercus robur*. Our results show that in all species, PST increased over time and that this change correlated most strongly with stand age, while the standardized precipitation–evapotranspiration index, a measure of drought, contributed to temporal trends in PST of *F. sylvatica* and *Q. robur*. Temporal variability of PST also increased over time in all species except *P. sylvestris*, while trends in temporal autocorrelation and among‐stand synchrony reflect species‐specific masting strategies. Our results suggest a pivotal role of plant ontogeny in driving not only the extent but also variability and synchrony of reproduction in temperate forest trees. In a time of increasing forest regrowth in Europe, we therefore call for increased attention to demographic effects such as aging on plant reproductive behavior, particularly in studies examining global change effects using long‐term time series data.

## Graphical abstract

Using 54 years of data for seven common temperate forest trees in Poland, we investigated how climate change and aging may affect temporal trends in mast‐seeding, measured by the change in extent, variability, autocorrelation, and synchrony of the proportion of seed‐producing trees (PST) over time. Contrary to expectations, changing climatic conditions only had a minor effect on temporal trends, while stand age was the dominant driver. Such demographic effects have previously been ignored but may play a major role in driving temporal changes of tree reproduction over time.

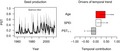

## 1 INTRODUCTION

Masting, the synchronized production of intermittent bumper crops in plant populations, is a global phenomenon with ecosystem‐wide consequences (Kelly & Sork, 2002; Koenig & Knops, 2000). The resulting resource pulses reverberate across trophic levels, driving population cycles from primary consumers to top predators and even pathogens such as Lyme disease (Bogdziewicz, Zwolak, & Crone, 2016; Jones, Ostfeld, Richard, Schauber, & Wolff, 1998; McShea, 2000; Ojeda & Chazarreta, 2018; Ostfeld & Keesing, 2000). A recent surge in masting research, fueled by the increased availability of long‐term time series datasets, has revealed that synchrony and variability of seed production are generally driven by a combination of internal resource dynamics of individuals coupled with population‐wide responses to annual variation in weather conditions preceding or during flowering and fruit maturation (Bogdziewicz, Steele, Marino, & Crone, 2018; Fernández‐Martínez, Vicca, Janssens, Espelta, & Peñuelas, 2017; Koenig, Knops, Carmen, & Pesendorfer, 2017; Pearse, Koenig, & Kelly, 2016; Pesendorfer, Koenig, Pearse, Knops, & Funk, 2016). Based on different proposed proximate mechanisms linking weather to seed production, climate change has been hypothesized to affect masting, but the direction and extent of the effects are unclear. Predicted effects range from negligible to extensive changes in the extent and variability of seed production in trees (Kelly et al., 2013; Koenig, Knops, Carmen, & Pearse, 2015; McKone, Kelly, & Lee, 1998). Importantly, a fundamental pattern underlying time series datasets has hitherto largely been ignored; regardless whether the data are based on stand‐level estimates or marked individuals, the age of the sampled trees increases over time.

Two recent studies that explicitly addressed the role of tree size, often the best proxy for age in field conditions, reported that the extent and temporal variability of seed production increased in larger trees, which suggests that aging may also be an important driver of masting behavior (Minor & Kobe, 2017, 2019). However, these studies presented comparative data from short time series, rather than tracking seed production throughout the process of aging, during which climatic conditions can change dramatically. Therefore, it remains an open question how aging and climate change contribute to temporal trends of mast‐seeding.

Older and larger trees generally produce greater seed crops, but the exact nature of the relationship between age and reproduction is poorly understood. In mature forests, aboveground net primary production (NPP) generally declines with stand age after a peak during early stand development, but does not stop completely in old growth forests (Gower, McMurtrie, & Murty, 1996; Luyssaert et al., 2008). This decline in growth efficiency is thought to arise from the imbalance of changes in photosynthetic area and increases in respiratory load as living stems accrue (Fernández‐Martínez et al., 2014; Ryan et al., 1997). In fast‐growing trees, a combination of declining gross primary production and changes in allocation may result in plateaus or slower scaling of NPP with age (Ryan, Binkley, Fownes, Giardina, & Senock, 2004). In the world's largest trees, relative growth can change over time and it shows little relationship to basal area, suggesting that allocation to growth or reproduction can remain flexible, even at advanced age and size (Sillett et al., 2015). Carbon allocation studies suggest that trees appear increase allocation to reproductive structures once the age of peak growth has been surpassed (Genet, Breda, & Dufrene, 2010; Hirayama, Nanami, Itoh, & Yamakura, 2008; Kozłowski, 1992; Thomas, 2011). Unsurprisingly, larger trees often produce larger seed crops, both in temperate and tropical communities (Minor & Kobe, 2017, 2019). Similarly, tree age is the strongest predictor of cone production in *Abies alba* and *Picea mariana* (Davi et al., 2016; Viglas, Brown, & Johnstone, 2013). Other work, however, suggests inconsistent patterns of seed production as a function of tree size (Greenberg, 2000; Greene & Johnson, 1994), hinting at stage‐ rather than age‐specific changes in reproductive strategies (Thomas, 2011).

While the hypothesis of age‐related increases in overall seed production seems intuitive as reproduction scales with size, the potential effects on the inter‐annual variability and synchrony in mast‐seeding trees have received little attention. The literature provides contradictory findings about the relationship between tree fecundity and the key elements of masting, temporal variability, autocorrelation, and large‐scale synchrony of seed production. In temperate forests, trees that produce large seed crops showed lower inter‐annual variability, stronger negative autocorrelation, and were more synchronous with the population mean than the general population (Minor & Kobe, 2017; Pesendorfer et al., 2016). Pearse et al. (2017) used data from 363 species of long‐lived iteroparous plants across the globe to show that the temporal variability of seed production has been increasing on a global scale over the last 100 years. The analysis did not explicitly account for age, but it was based on time series datasets. The only significant predictors of temporal trends in variability were the general masting tendency of the species (mean temporal variability over the whole time series) and the declining long‐term mean in seed production, but not latitude, temperature increase, or nitrogen deposition (Pearse et al., 2017). Somewhat surprisingly, considering that variation in seed production is often directly or indirectly associated with abiotic conditions, the study concludes that factors other than climate change may drive the observed long‐term increase in variability.

Climate change effects on masting seem like a foregone conclusion because of the strong relationship between weather and seed production patterns, but mechanistic models provide a more complicated picture. In addition to a large number of studies that reported direct effects of variation in abiotic conditions on flowering and fruit development, some research suggests that differential cues, for example, the difference between mean summer temperatures of the two preceding years (Δ*T* = *T*_‐2_ – *T*_‐1_) best predict seed production in a subset of masting species (Kelly et al., 2013; Kon & Saito, 2015; Nussbaumer et al., 2018; Pearse et al., 2016). Importantly, the latter mechanism would be insensitive to increases in mean temperature predicted by global change models. Several studies subsequently challenged this assertion, either by showing that the cue Δ*T* is closely correlated with proximate drivers of seed production (Pearse, Koenig, & Knops, 2014) or by showing that mechanistic models of “resource‐limited floral induction,” that is, the interaction between resource dynamics (driven by *T*_‐2_) and abiotic conditions at *T*_‐1_, result in the same relationship of masting and Δ*T* for several of the species in the Kelly et al. (2013) study (Monks, Monks, & Tanentzap, 2016). In addition to rising temperatures, other anthropogenic factors such as increased nitrogen deposition or atmospheric CO_2_ concentrations, which potentially decrease plant nutrient concentrations and fruit production, could also affect masting patterns over time (Bogdziewicz, Crone, Steele, & Zwolak, 2017; Fernández‐Martínez, Vicca, Janssens, Ciais, et al., 2017; Peñuelas et al., 2013). Potential climate change effects on mast‐seeding therefore remain a point of contention.

The aim of this study was to contrast the predictions of the climate change and aging hypotheses by determining the contribution of climate change and stand age to temporal trends in the extent, temporal variability, and large‐scale synchrony of seed production (Table 1). We took advantage of a 54 year long dataset on the proportion of seed‐producing trees (“PST” hereafter) in seven common European forest trees across Poland: European silver fir *A. alba* Mill., European larch *Larix decidua* Mill., Norway spruce *Picea abies* (L.) H. Karst, Scots pine *Pinus sylvestris* L., sessile oak *Quercus petraea* (Matt.) Liebl., pedunculate oak *Quercus robur* L., and European beech *Fagus sylvatica* L. First, we investigated whether PST increased over the study period and what may have caused the observed changes over time. Specifically, we tested whether increasing stand age or changes in abiotic conditions, such as temperature, precipitation, or drought best predicted the observed increase in PST. In a second set of analyses, we then determined how temporal changes of the significant predictors contributed to temporal trends of PST (Fernández‐Martínez et al., 2019). Based on the literature on reproductive allocation and intraspecific differences in masting strategies, we predicted that PST and its variability would increase with stand age, while climate effects would show species‐specific variation that reflect the relationship between weather, flowering dynamics, and PST.

**Table 1:** Terminology and definitions of variables considered in study

Variable (acronym)	Description	Values
Proportion of seed‐producing trees (PST)	Annual estimates of the proportion of fructified trees for each species in each site, to the nearest 0.1	0–1
PST in previous year (PST1)	The PST value from the previous year is used to account for temporal autocorrelation in generalized linear mixed models of PST	0–1
Temporal variability (PV)	Inter‐annual variability of PST values, calculated as average proportional difference between all combinations of observed values (proportional variability index—PV), calculated for time series or using 10 year moving window	0–1
Temporal autocorrelation (AR1)	Lag‐1 autocorrelation of PST values for species and site, calculated for overall time series or using 10 year moving window	−1 to 1
Among‐site synchrony (*r*_s_)	Mean Spearman's rank correlation coefficient among time series for each species, calculated in 10 year moving windows	0–1
Site	Regional Forest Directorates (see Table S1)	*N* = 17
Stand age	Mean age of a species' trees population at individual sites	50–70
Temporal trend	Change of parameter values over time: standardized parameter estimate (*β*) from (generalized) linear mixed models with the fixed effect “year” and random effect “site”	
Temporal contribution	Effect of temporal trends in predictors on temporal trend in masting parameters, estimated by contrasting models with focal predictor fixed at median value to models (Fernández‐Martínez & Maspons, 2019)	

## 2 MATERIALS AND METHODS

### 2.1 Seed production and stand age data

Data on PST for each focal species for the years 1958–2012 were obtained from the Polish General Directorate of State Forests (Kantorowicz, 2000). In annual reports starting in 1951, each of the 17 Polish Regional Forest Directorates (hereafter “sites”; Table S1) estimates the percentage of trees that fructified (to the nearest 10%) in a site in a given year (Kantorowicz, 2000). Here, we use the estimates converted to proportions of PST as a broad measure for the level of seed production. PST essentially captures the stand‐level synchrony of seed production, a factor that is strongly correlated with landscape levels of seed production (Koenig et al., 2003). Because weather and stand age data were only available for all sites starting in 1958, we did not use the previous years of data. A previous study on a subset of species (*Quercus* spp. and *F. sylvatica*) and shorter time series (17 years) found that PST estimates correlate strongly (*r* > .80) with the mass of seeds collected from the forest floor and seed traps by Regional Forest Directorates for the Polish Forest Gene Bank (Bogdziewicz, Szymkowiak, et al., 2017). Similar comparisons are not available for the other species in the study.

Stand age data, which are determined by Polish State Forests based on the planting calendars and by coring trees, were obtained for each species and each site from the Polish National Statistical Office (http://stat.gov.pl/). The Statistical Office publishes the data in annual reports that consist of forest cover in hectares belonging to five age classes (class I: age ranging 1–20; II: 21–40; III: 41–60; IV: 61–80; V: >81). This national‐scale data is provided separately for deciduous and coniferous species. Based on that data, we calculated the mean forest age for angiosperms and conifers for each site and year. Because the age data were not available for all years with seed production data, we inter‐ and extrapolated partial trends in time for both gymnosperms and angiosperms from 1958 to 1966 and from 2009 to 2012 (Figure S1). Given that stand age dynamics of angiosperms were well correlated with stand age of all forests, we also used stand age from all forests to interpolate the gaps in stand age time series of angiosperms species (Figure S1). Data on *L. decidua* were only available until 2010.

### 2.2 Weather data

Weather data for the year before the fruiting period (October–December) were obtained from the Institute of Meteorology and Water Management in Poland. For each site, we obtained data from the nearest meteorological station (as measured to the center; mean distance = 33 km, range = 7–59 km) for which the continuous records of daily total precipitation and mean temperature were available for the study period. Seasonal temperature (*T*) and precipitation parameters (*P*) were calculated by averaging daily measurements for winter (*T*_wi_, *P*_wi_; December–February), spring (*T*_sp_, *P*_sp_; March–May), summer (*T*_su_, *P*_su_; June–August), and autumn (*T*_au_, *P*_au_; September–November) periods. Furthermore, we obtained the standardized precipitation–evapotranspiration index (SPEI) from May to October as a measure of atmospheric hydric conditions during the growing period (Vicente‐Serrano, Begueria, & Lopez‐Moreno, 2010). The SPEI is calculated from the difference of daily values of precipitation and potential evapotranspiration (Beguería & Vicente‐Serrano, 2013). High SPEI values therefore indicate wet conditions, while low values are indicative of drought.

### 2.3 Statistical analyses

#### 2.3.1 Temporal trends and drivers of PST

To determine whether PST changed over time (i.e., its temporal trend; Table 1), we constructed generalized linear mixed models (GLMM) with binomial error distributions. The models contained the fixed effect “year” and the random effect “site” to account for repeated measurements. We used the standardized slope estimate (*β*) of PST over time as the estimate for its temporal trend (Table 1).

We used model selection and averaging to determine which variables best predicted PST per site and year, then we determined how temporal changes in the significant predictors (e.g., the increase in drought severity) contributed to the temporal trend of PST. To do so, we first fitted global (saturated) GLMMs of the binomial family for PST (Appendix S1). We included the fixed effects stand age, climate parameters for the four seasons preceding the focal year's fall (the time at which seed crops were estimated), SPEI, the PST of the previous year (PST1) to account for temporal autocorrelation, as well as its first‐order interaction with stand age (stand age: PST1) to account for potential changes in temporal autocorrelation with age (Table 1). We then used model selection based on AIC to determine the abiotic variables that best predicted PST by considering all potential submodels. Because no dominant submodel (model weight *w*_i_ > 0.9) emerged for any of the species, we used model averaging for all the models within ΔAIC < 2 to estimate variable importance, standardized parameter estimates *β*, their standard error, and associated *z*‐ and *p*‐values for the fixed effects. Details for model selection and averaging are presented in Appendix S1.

In the second step, we estimated the temporal contributions of changes in climate, stand age, and temporal autocorrelation to the temporal trend in PST for each studied species (Table 1). We used the function TempCont in the R package “TempCont” (Fernández‐Martínez & Maspons, 2019), which first extracts the observed temporal trend in annual PST using GLMMs with an auto‐regressive and moving average (*p* = 1; *q* = 0) correlation structure to account for temporal autocorrelation. Then, it calculates the trend of PST predicted by the final model and the trend of PST predicted by the same model while maintaining the temporally varying predictors constant one at a time (e.g., spring temperature *T*_sp_ was held constant using the median per site, while all other predictors varied according to the observations). The difference between the prediction of the final model and the prediction of the model when one predictor was controlled is considered the contribution of that controlled variable to the temporal change in PST (Fernández‐Martínez & Maspons, 2019). For this analysis, we only considered significant predictors (*p* < .05) of annual variation in PST.

#### 2.3.2 Variability and synchrony of fruiting trees

To assess how temporal variability (PV) and lag‐1 autocorrelation (AR1) of the proportion of PST changed over time, we calculated their values in 10 year moving windows from 1958 to 2012 for every site and species. PV, the proportional variability index, consists of the average percent difference between all combinations of observed values. This measure is preferable to mean‐based measures of variability, such as the coefficient of variation, as it is truly proportional across the parameter range and does not exhibit pathological issues at the extremes (Heath, 2006; Heath & Borowski, 2013). Because values derived from sliding‐window averages are highly temporally autocorrelated, we calculated temporal trends of mean PV and AR1 for each species (across sites) using the Theil–Sen's slope estimator, which is considered robust against non‐normally distributed data and temporal autocorrelation (Ohlson & Kim, 2015). We used average trends across sites for each species because mixed models of multiple time series do not perform well with autocorrelation terms associated with random effects (Zuur, Ieno, Walker, Saveliev, & Smith, 2009).

Finally, to determine whether among‐site synchrony within species changed with time and whether a similar pattern was evident in the significant abiotic predictors of PST, we calculated the mean Spearman's rank correlation (*r*_s_) for PST values and weather predictors among the 17 sites using a 10 year moving window basis for each species and then used Theil–Sen's slopes to test whether there was a significant change over time.

All analyses were conducted in R, version 3.6.1 (R Core Team, 2018). We used the packages “glmmTMB,” version 0.2.3 (Magnusson et al., 2019) to construct GLMMs, “DHARMa,” version 0.2.4 (Hartig, 2019) for model checking and diagnostics, “MuMIn,” version 1.43.6 (Barton, 2019) to conduct model selection and averaging, “mblm,” version 0.12.1 (Komsta & Komsta, 2013) to estimate Theil–Sen slopes, and “TempCont,” version 0.1.0 (Fernández‐Martínez & Maspons, 2019) to estimate the temporal contribution of significant fixed effects. To allow direct comparison among variables, all predictors were standardized by centering and scaling by dividing by their standard deviation (Zuur et al., 2009).

## 3 RESULTS

### 3.1 Temporal trends of seed production metrics

**Figure 1:**
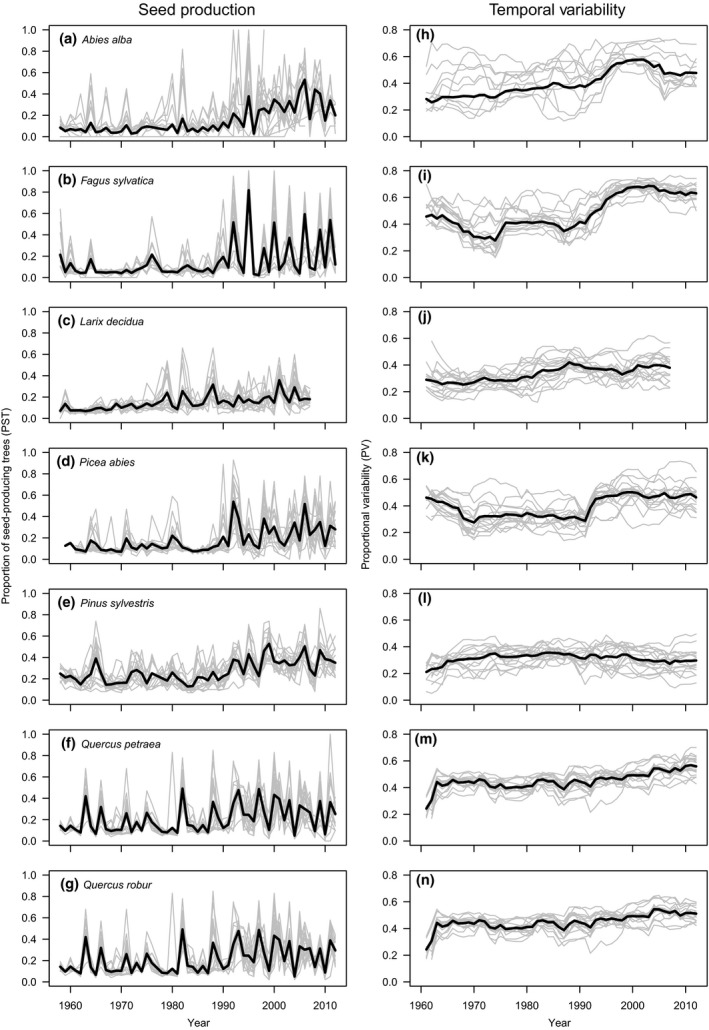
Time series of the (a–g) proportion of seed‐producing trees (PST) and (h–n) its temporal variability (PV) for temperate forest stands in Poland from 1958 to 2012. PV was calculated using 10 year moving windows. Gray lines indicate time series for 17 sites, black lines indicate species mean

**Figure 2:**
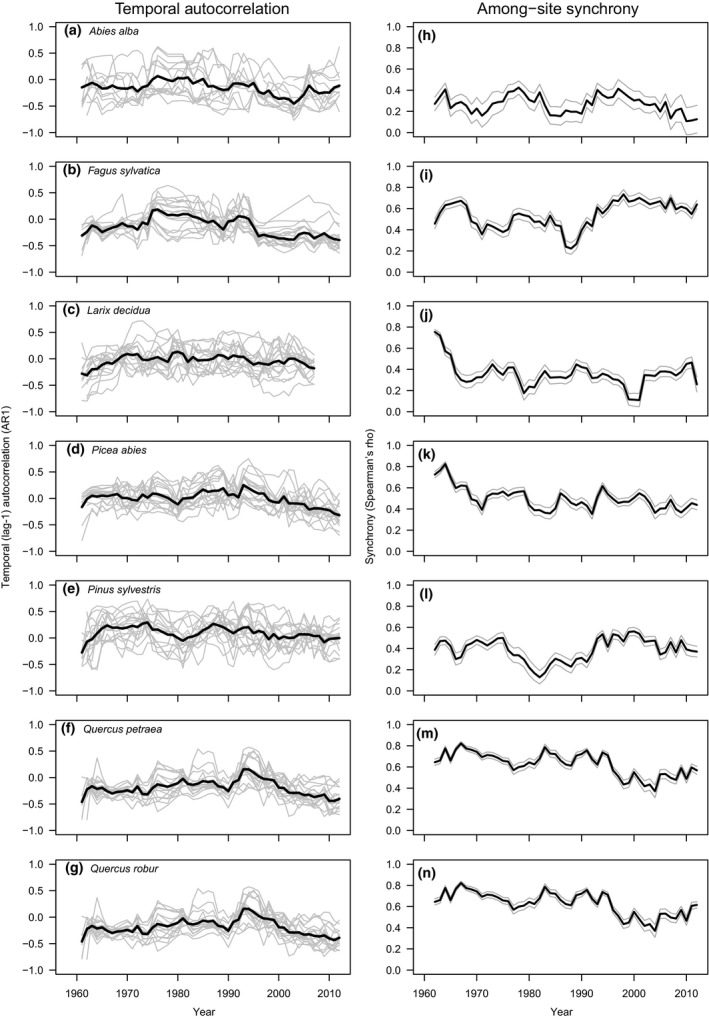
(a–g) Lag‐1 temporal autocorrelation (AR1) and (h–n) among‐site synchrony (*r*_s_) of the proportion of seed‐producing trees for seven temperate forest tree species in Poland from 1958 to 2012, calculated using 10 year moving windows. In (a–g), gray lines indicate values for 17 sites, in (h–n) gray lines indicate 95% confidence interval; black lines indicate species mean

In all seven tree species, the PST increased over time, thus showing a positive temporal trend (Figure 1a–g; Table 2a). PST in *A. alba* showed the strongest temporal increase (*β* = 0.7), while the slowest increase was observed in *L. decidua*,* Q. petraea*, and *Q. robur* (*β* = 0.3; Table 2a). *F. sylvatica* exhibited the largest interannual variability (PV) in PST, while *P. sylvestris* exhibited the lowest variability (Figure 1h–n; Table 2b). PV showed positive temporal trends in all species except *P. sylvestris* (Table 2b). Temporal autocorrelation (AR1) was similar among species, with *F. sylvatica* exhibiting the strongest negative autocorrelation and *P. sylvestris* showing slightly positive autocorrelation in PST (Figure 2a–g; Table 2c). AR1 became increasingly negative over time in *A. alba*,* F. sylvatica*, and *P. abies*, but showed no temporal trend in the other species. Synchrony (*r*_s_) among sites was highest for the two *Quercus* species, while *A. alba* was the species with the lowest synchrony among sites (Table 2d). Synchrony increased only in *F. sylvatica* over the study period and declined in *L. decidua*,* P. abies*, and the *Quercus* species. (Figure 2h–n; Table 2d).

**Table 2:** Masting metrics and their temporal trends for seven temperate forest species in Poland from 1954 to 2012. (a) The proportion of seed‐producing trees (PST), (b) temporal variability PV, (c) temporal autocorrelation AR1, and (c) large‐scale synchrony *r*_s_. Means (±*SE*) as well as temporal trends (*β*) standardized parameter estimates (±*SE*), *z*‐ and *p*‐values for (G)LMMs that regress values against year, all with random effect “site”

(a) Proportion of Seed‐Producing Trees (PST)						
Species	Mean	*SE*	Trend	*SE*	*z*	*p*
*Abies alba*	13.143	0.557	**0.701**	**0.115**	**6.1**	<.001
*Fagus sylvatica*	14.098	0.621	**0.557**	**0.103**	**5.4**	<.001
*Larix decidua*	14.669	0.303	**0.331**	**0.102**	**3.3**	.001
*Picea abies*	18.504	0.481	**0.452**	**0.095**	**4.8**	<.001
*Pinus sylvestris*	27.031	0.419	**0.332**	**0.078**	**4.3**	<.001
*Q. petraea*	20.338	0.499	**0.297**	**0.085**	**3.5**	<.001
*Quercus robur*	20.555	0.487	**0.315**	**0.085**	**3.7**	<.001

(b) Temporal variability (PV)						
Species	Mean	*SE*	Trend	*SE*	*t*	*p*
*A. alba*	0.447	0.040	**0.034**	**0.004**	**8.1**	<.001
*F. sylvatica*	0.516	0.021	**0.011**	**0.001**	**9.2**	<.001
*L. decidua*	0.393	0.008	**0.003**	**0.001**	**11.5**	<.001
*P. abies*	0.455	0.007	**0.003**	**0.001**	**4.9**	<.001
*P. sylvestris*	0.375	0.010	0	0	1.1	.637
*Q. petraea*	0.482	0.005	**0.003**	**0.001**	**8.1**	<.001
*Q. robur*	0.476	0.004	**0.002**	**0.001**	**7.9**	<.001

(c) Lag‐1 temporal autocorrelation (AR1)						
Species	Mean	*SE*	Trend	*SE*	*t*	*p*
*A. alba*	−0.011	0.014	**−0.003**	**0.001**	**−3.9**	<.001
*F. sylvatica*	−0.057	0.021	**−0.005**	**0.001**	**−3.8**	.001
*L. decidua*	−0.038	0.024	0.001	0.001	0.5	.611
*P. abies*	−0.013	0.026	**−0.004**	**0.001**	**−3.4**	.001
*P. sylvestris*	0.044	0.027	−0.003	0.001	−1.9	.062
*Q. petraea*	0.001	0.025	−0.001	0.001	−0.4	.568
*Q. robur*	−0.045	0.023	−0.001	0.001	−0.3	.546

(d) Among‐site synchrony (*r*)						
Species	Mean	*SE*	Trend	*SE*	*z*	*p*
*A. alba*	0.376	0.018	−0.001	0.001	−1.7	.092
*F. sylvatica*	0.567	0.015	**0.003**	**0.001**	**2.7**	.010
*L. decidua*	0.496	0.012	**−0.002**	**0.001**	**−2.6**	.012
*P. abies*	0.610	0.011	**−0.004**	**0.001**	**−5.2**	<.001
*P. sylvestris*	0.563	0.011	0.001	0.001	1.1	.266
*Quercus petraea*	0.687	0.011	**−0.005**	**0.001**	**−6.9**	<.001
*Q. robur*	0.707	0.008	**−0.005**	**0.001**	**−6.7**	<.001

Bold values indicate significant temporal trends.

In both gymnosperm and angiosperm species, the mean age increased in parallel since the late 1950s when the mean equaled ca. 55 years in coniferous forests and 52 in angiosperm forests, eventually reaching ca. 63 years in 2012 (Figure S1). During that time, the forest covered by trees older than 60 years grew from ~2,217,000 ha (36% of total cover) to ~2,904,000 ha (44% of total cover), while the share of the youngest class (below 20 years) decreased from 21% to 12%.

### 3.2 Temporal change and controls of PST

**Figure 3:**
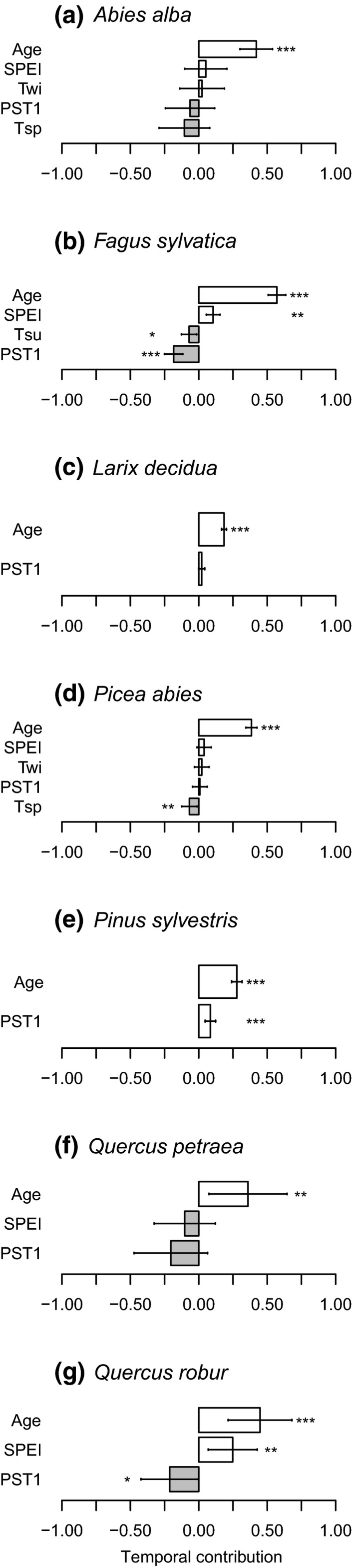
Temporal contribution of stand age, abiotic predictors, and proportion of seed‐producing trees in previous year (PST1) to temporal trends in PST of seven European forest species in Poland. Only significant predictors of annual values are included in trend models (a–g). Error bars indicate standard errors. See Section 2 for variable definitions and Appendix S1 for model selection tables. SPEI, standardized precipitation–evapotranspiration index. Significance levels: *p < .05; **p < .01; ***p < .001

Of the factors examined, stand age correlated most strongly with annual PST in all species. For all species, model selection and averaging revealed that stand age showed high variable importance and was a significant predictor of PST (see Appendix S1 for full model selection results). Age was the only significant predictor for *A. alba* (*β* = 0.59 ± 0.12; *z* = 5.0; *p* < .001; Table S3) and *L. decidua* (*β* = 0.16 ± 0.07; *z* = 2.2; *p* = .026; Table S9), and in both species, it also contributed significantly to the temporal trend of PST (Figure 3a,c; Tables S4 and S10). The drought index SPEI was a negative predictor of PST in *F. sylvatica* (*β* = −0.42 ± 0.13; *z* = 3.1; *p* = .002; Table S6), *Q. petraea* (*β* = −0.28 ± 0.10; *z* = 2.8; *p* = .005; Table S18), and *Q. robur* (*β* = −0.29 ± 0.10; *z* = 3.0; *p* = .002; Table S21), indicating that seed production in these species is reduced following summer droughts. In *F. sylvatica* and *Q. robur*, SPEI also contributed to the temporal trend of PST (Figure 3b,g; Tables S7 and S22). In *P. abies*, spring temperatures (*T*_sp_) correlated negatively with PST (*β* = −0.26 ± 0.12; *z* = 2.0; *p* = .041; Table S12) and there was a significant effect of changing temporal autocorrelation with age (stand age: PST1: *β* = −0.19 ± 0.09; *z* = 2.1; *p* = .034; Table S12), but neither contributed significantly to temporal trends in PST (Figure 3d; Table S13). Stand age (*β* = 0.24 ± 0.09; *z* = 2.7; *p* = .007; Table S17) and PST1 (*β* = 0.25 ± 0.09; *z* = 2.8; *p* = .005; Table S15) were the only significant predictors of PST in *P. sylvestris*, and also contributed to its temporal trend (Figure 3e; Table S16).

### 3.3 Trends in temporal variability, autocorrelation, and synchrony in seed production

Temporal variability (PV) of PST was highest in *F. sylvatica*, followed by the *Quercus* species, and was lowest in *P. sylvestris* (Figure 1a–g; Table 2b). Similarly, PV increased over time in all species except *P. sylvestris*, where it remained constant over the study period (Figure 1h–n; Table 2b). In parallel, the autocorrelation in seed production (AR1) became increasingly negative in all species, except the two *Quercus* species and *L. decidua*, where it remained constant after an initial increase (Figure 2a–g; Table 2c). Synchrony in seed production decreased during the study period in *Q. petraea*,* Q. robur*, and marginally in *A. abies*, increased in *F. sylvatica*, and remained unchanged in *L. decidua* and *P. sylvestris* (Figure 2h–n; Table 2d). The time series trends in synchrony with weather variables showed that spring precipitation was the only weather variable presenting a significant negative trend that could be related to the decrease in synchrony in seed production (Table S23). Instead, winter and spring temperatures presented positive trends in their synchrony.

## 4 DISCUSSION

Our analysis of long‐term masting patterns supports the hypothesis that stand age plays a major role in determining the extent and variability of the proportion of seed‐producing trees (PST), a coarse measure of mast‐seeding in European temperate forest trees. As the mean PST increased over the 54 year study period in stands of all seven species, so did the temporal variability except in *P. sylvestris* (Figure 1). We further found that temporal trends in stand age, not abiotic parameters most affected by climate change, contributed most to the observed increase in PST over time (Figure 3). External drivers, such as changes in temperature and precipitation, had a much weaker relationship with the trends in the annual extent of seed production. This suggests that ontogeny is an important driver of temporal changes in masting behavior and highlights that a change in the age structure through management may influence the extent of this reproductive behavior.

Our findings of small effects of changes in abiotic conditions on trends in PST still provide some support for the climate change hypothesis, as they fit well with the recent findings on proximate mechanisms underlying masting. A growing body of literature suggests that weather effects on mast‐seeding are not as direct as originally thought and may vary across the geographic range of species (Bogdziewicz, Szymkowiak, Fernández‐Martínez, Peñuelas, & Espelta, 2019; Nussbaumer et al., 2018). Weather drives resource budgets of individual plants not only by modulating annual resource acquisition (Bogdziewicz, Szymkowiak, et al., 2017; Fernández‐Martínez, Garbulsky, Peñuelas, Peguero, & Espelta, 2015; Smaill, Clinton, Allen, & Davis, 2011) but also flowering and seed maturation dynamics which create population‐wide patterns of seed production and its synchrony (Abe et al., 2016; Bogdziewicz, Szymkowiak, et al., 2017; Koenig et al., 2015; Pearse et al., 2017; Pesendorfer et al., 2016; Venner et al., 2016). Because these abiotic effects can be nonlinear and often take the form of correlated crop failure (Bogdziewicz et al., 2018; Fernández‐Martínez, Bogdziewicz, Espelta, & Peñuelas, 2017), extrapolations to climate change effects are challenging (e.g., Kelly et al., 2013; Koenig et al., 2015). In fact, a meta‐analysis of mast‐seeding studies on a subset of the species included in this study (excluding *A. abies* and *L. decidua*) showed that only for *F. sylvatica* and to a limited degree for *P. abies*, the same weather variables correlated with seed production across different geographic regions in Europe (Nussbaumer et al., 2018).

We anticipate that future research with increasingly detailed studies of long‐term time series and experiments addressing hypothesized mechanisms will improve our understanding of the internal and external drivers of masting patterns in plant populations. Like the current study, the majority of masting research is based on correlations rather than experiments and the metrics of seed production often vary between study systems (Bogdziewicz et al., 2020). The measure of seed production used here, the PST, only captures a very broad aspect of masting as it does not estimate the actual number of seeds produced per area, which can be dominated by few individuals on a neighborhood scale (Minor & Kobe, 2017). However, on a landscape scale, PST captures the essential reproductive step for trees in a given year—whether to produce seeds or not. This provides the fundamental first step of a sequence of factors that determine the ultimate number of viable seeds on trees (Pearse et al., 2016). In standardized forest monitoring programs, such as the International Cooperative Programme on Assessment and Monitoring of Air Pollution Effects on Forests, PST would cover all categories of individual‐level fructification (“scarce,” “common,” “abundant”) except “absent” (UNECE ICP, 2016). However, PST will be particularly useful in systems with low per‐capita seed production, where it captures the majority of variation in annual seed production.

Temporal autocorrelation, a measure that is thought to reflect resource dynamics of trees because they may not be able to produce a large crop for consecutive years, generally declined over time; it decreased significantly in *A. alba*,* P. abies*, and *F. sylvatica* (Figure 2; Table 2c). In *F. sylvatica* and *P. abies*, this appears to be a consequence of aging, as indicated by the significant interaction between PST1 and age (Tables S6 and S12). This result is somewhat surprising as the extent of stand‐level mean autocorrelation was considerably less negative than in smaller populations or individual trees, likely a consequence of the coarse measure used (Koenig et al., 2003; Sork, Bramble, & Sexton, 1993). As trees age and produce larger crops, they may become more resource limited when balancing maintenance and reproduction, as essential compounds are increasingly depleted for the subsequent year (Pesendorfer et al., 2016).

We also found changes in large‐scale spatial synchrony in several species: synchrony among stands decreased in *L. decidua*,* Q. petraea*,* Q. robur*, and *P. abies*, while it increased in *F. sylvatica* (Figure. 2). Weather is certainly involved in driving large‐scale synchrony of reproduction in masting plants through the Moran effect—the fact that weather is spatially autocorrelated over large areas (Koenig & Knops, 2001; Pearse et al., 2016; Schauber et al., 2002)—but the exact mechanisms are likely species‐specific and depend on how abiotic conditions affect reproduction in different taxa (Bogdziewicz, Szymkowiak, et al., 2017; Koenig et al., 2016). In oaks, temperature and rainfall in spring strongly modulate crop size, through affecting phenological synchrony of flowering and pollination efficiency. As large‐scale spatial synchrony of precipitation decreased over last 50 years (Table S2), it possibly contributed to the decreased synchrony in *Quercus* species (Bogdziewicz, Crone, et al., 2017; Koenig et al., 2015; Schermer et al., 2019). Interestingly, decreased synchrony in oaks might have important ecological consequences by dampening the predator satiation effect, the most broadly supported selective advantage of masting in these species (Kelly & Sork, 2002). The high synchrony in acorn production starves predators during years of low or absent reproduction and satiates them during high reproduction years, leaving large numbers of seeds intact (Espelta, Cortés, Molowny‐Horas, Sánchez‐Humanes, & Retana, 2008). Therefore, a scenario of increasing acorn production coupled with a lower among‐site synchrony, as the one reported here, would favor more intense acorn predation, particularly by highly mobile predators (e.g., *Cydia* sp moths; Ruiz‐Carbayo, Bonal, Pino, & Espelta, 2018).

In contrast, the large‐scale reproductive synchrony in *F. sylvatica* increased, despite unchanged synchrony in SPEI, the only significant correlate of PST in this study, and stable synchrony in summer temperatures, the strongest predictor of large flowering and seed production efforts in other areas of Europe (Piovesan & Adams, 2001; Vacchiano et al., 2017). This suggests that factors other than climate or aging may drive this trend. For example, changes in pollen transfer efficiency among disparate populations may affect synchrony over large geographical regions (Satake & Iwasa, 2000, 2002). Conversely to oaks, whatever the factor involved in increased synchrony in beech, this would reinforce the benefits of masting for this species to escape seed predation by highly mobile predators (Nilsson & Wästljund, 1987).

Future work should aim to connect dynamics at smaller spatial scales to the large‐scale patterns observed here. While we are confident that the data allowed us to capture biologically relevant changes of seed production patterns, due to both the extensive temporal and spatial scales involved in this study, as well as parallel trends in distantly related species, other factors could contribute to the observed patterns. In addition to age, other relevant aspects of stand dynamics may change over long time periods, which we have not considered here. For example, the demographic composition, stand density, and competition with other species may all shift over multiple decades, so that resource and pollination dynamics underlying seed production could be impacted. Further limitations of our study include the coarse measurement of seed production levels which obscures the contribution of super‐producers or reproductively inhibited trees (Minor & Kobe, 2017). Similarly, impacts of herbivores and seed predators, whose population dynamics may be affected by the observed increases in drought frequency and duration, could not be assessed here (Bogdziewicz et al., 2016). Changes in nitrogen deposition or atmospheric CO_2_ concentrations, which potentially affect plant nutrient concentrations and fruit production (Bogdziewicz, Crone, et al., 2017; Fernández‐Martínez, Vicca, Janssens, Ciais, et al., 2017; Peñuelas et al., 2013) were beyond the scope of this paper, in which we focus on drought, temperature, and precipitation. We were also unable to assess the impact of changing management or sampling decisions during data collection by Polish authorities.

### 4.1 Species differences reflect alternate proximate mechanisms of masting

Increased drought severity, identified by decreased SPEI, had a negative effect on reproduction trends in all the three angiosperm species (*Q. petraea*,* Q. robur*,* F. sylvatica*). Over the study period, the frequency and spatial extent of drought in Poland have increased strongly, though they are quite variable from year to year (Somorowska, 2016). Drought has previously been identified as an important proximate driver of masting in other *Quercus* species (Espelta et al., 2008; Fernández‐Martínez, Belmont, & Espelta, 2012; Koenig et al., 2016). For *F. sylvatica*, previous work suggests a less prominent and more localized role of drought in shaping annual seed production (Nussbaumer et al., 2018; Vacchiano et al., 2017), yet drought (SPEI) was the only significant abiotic predictor in our study for that species. Projected increases in drought intensity will thus likely have detrimental effects on seed production in angiosperm tree species.

The results from the gymnosperm species provide a more complex picture. While the mean proportion of PST in all four species increased with stand age, we found no effect of changes in abiotic conditions in *A. alba*,* L. decidua,* and *P. sylvestris* (Table 2a; Figure 3). In *P. abies*, spring temperature was the strongest negative predictor of PST trends apart from age, suggesting that flowering dynamics may be affected. Previous work has shown that annual seed production levels are generally correlated with summer temperatures in the previous two years, but the species shows relatively low interannual variation in seed production (Selås, Piovesan, Adams, & Bernabei, 2002). In fact, *P. sylvestris* was the only species that did not show increased proportional variability over time while maintaining positive autocorrelation values throughout (Figure 1; Table 2b). These results provide support to the recent suggestion that *P. sylvestris* and *L. decidua* may differ fundamentally from other masting species, the former because it has lower temporal variability in seed prediction and the latter because it is the only deciduous conifer (Bisi et al., 2016). Climate change effects on the reproduction of these two species may thus be minimal.

In summary, we found that over the last five decades, changes in stand age correlated much stronger with temporal trends in the PST than changing abiotic conditions, despite a dramatic increase in drought episodes. This supports the hypothesis that plant ontogeny is a strong driver of masting and calls for increased attention to demographic effects on plant reproductive behavior which has largely been overlooked (Koenig et al., 2017; Pesendorfer, Bogdziewicz, Koenig, Ledwoń, & Żywiec, 2019; Thomas, 2011). Considerable research effort has been invested into understanding how masting trees will respond to ongoing global change (Allen, Hurst, Portier, & Richardson, 2014; Bogdziewicz et al., 2019; Buechling, Martin, Canham, Shepperd, & Battaglia, 2016; Crone & Rapp, 2014; Kelly et al., 2013; Koenig et al., 2015; McKone et al., 1998; Monks et al., 2016; Pérez‐Ramos, Ourcival, Limousin, & Rambal, 2010; Pearse, LaMontagne, & Koenig, 2017), due to anticipated profound consequences for plants, their herbivores, and even more distantly connected taxa (Jones et al., 1998; Ostfeld & Keesing, 2000). Although we still know little about how exactly variability of reproduction will change in response to warming or N‐fertilization, our study implies that age‐related intensification of masting may be of at least similar magnitude and thus importance. Ultimately, our results suggest that such long‐term oscillation in variability can be a normal feature of aging forests recovering from overexploitation, as is the case for Polish forests and many other areas in Europe and the Northern Hemisphere (Song et al., 2018).

## CONFLICT OF INTEREST

The authors declare no competing interest.

## Data Availability

The data used in this manuscript are available upon request from the authors.
