# Supplementary material for: Investigating the relationship between climate, stand age, and temporal trends in masting behavior of European forest trees
Source: Glob Chang Biol. 2020 Jan 17;26(3):1654–67. doi: 10.1111/gcb.14945 (PMC7079002; doi:10.1111/gcb.14945)
Supplement: Supplementary file 1 [file GCB-26-1654-s001.docx]

## Supporting Information

Article title: **Investigating the relationship between climate, stand age, and temporal masting trends of European forest trees**

Authors: **Mario B. Pesendorfer, Michał Bogdziewicz, Jakub Szymkowiak, Zbigniew Borowski, Władysław Kantorowicz, Josep M. Espelta, Marcos** **Fernández-Martínez**


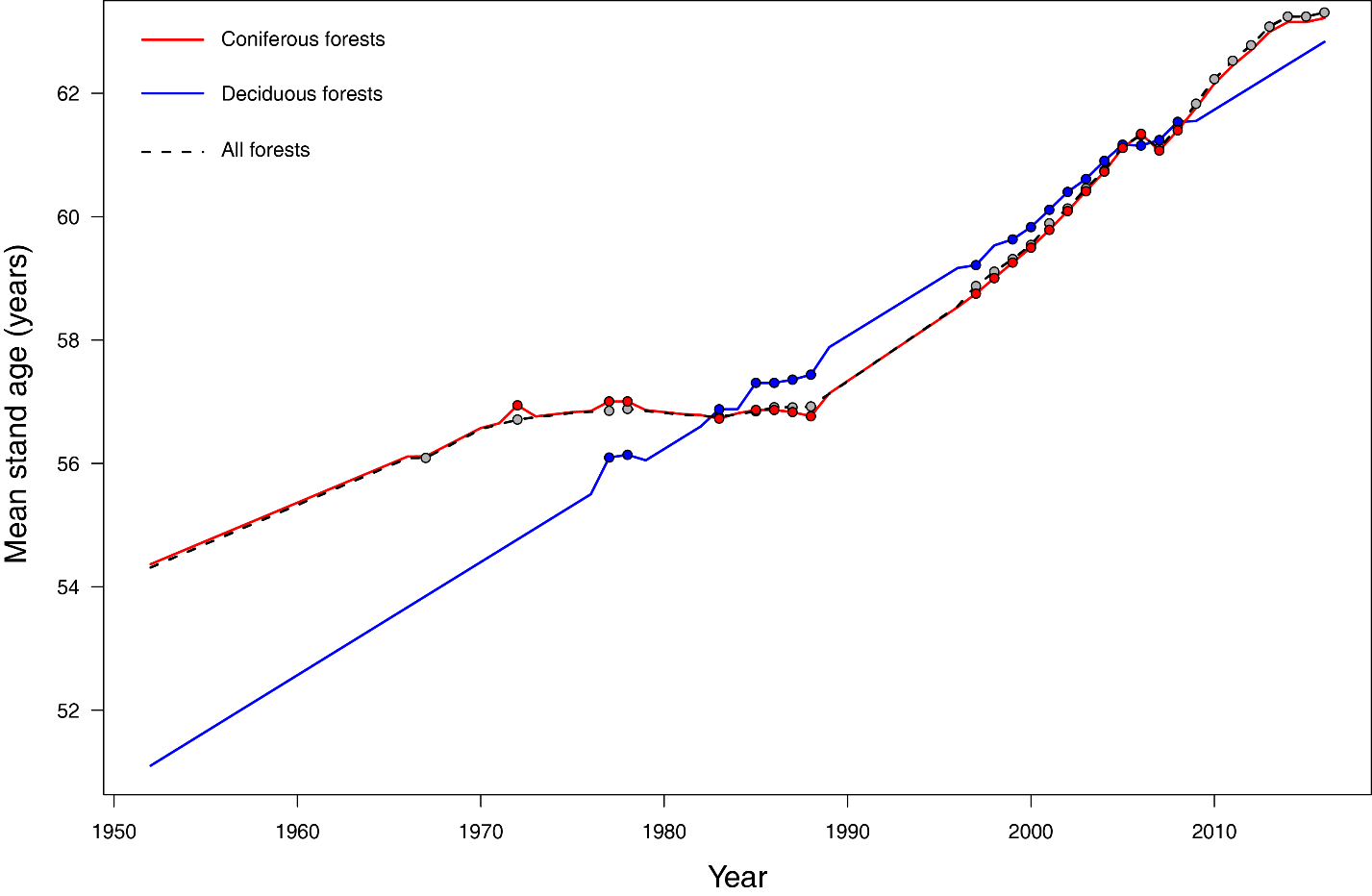
**Figure S1** Stand age dynamics over time. Red and blue solid lines show inter- and extrapolated stand age, respectively, for coniferous and deciduous forests. The dashed black line indicates stand age evolution of all forests altogether. Dots show published estimates of stand age for coniferous, deciduous and all forests (red, blue and grey dots, respectively).

**Table S1: Species composition of 17 Polish Regional Forest Directorates (sites).**
Area, in hectares, dominated by each species (with the exception for *L. decidua*, for which this information is not available). Reported on January 1^st^, 2018.

| **Site** | ***A. alba*** | ***F. sylvatica*** | ***P. abies*** | ***P. sylvestris*** | ***Quercus*** | **Total** |
| --- | --- | --- | --- | --- | --- | --- |
| BIAŁYSTOK | 12 | 63 | 77,132 | 521,039 | 58,836 | 840,561 |
| GDAŃSK | 234 | 46,287 | 7,465 | 239,035 | 19,011 | 341,581 |
| KATOWICE | 21,713 | 61,302 | 56,642 | 471,456 | 60,014 | 747,913 |
| KRAKÓW | 94,395 | 79,639 | 62,869 | 88,452 | 24,368 | 379,746 |
| KROSNO | 98,938 | 153,282 | 17,700 | 198,549 | 31,501 | 590,746 |
| LUBLIN | 12,610 | 18,899 | 2,501 | 437,222 | 82,976 | 676,487 |
| ŁÓDŹ | 1,642 | 2,635 | 896 | 363,746 | 26,701 | 445,785 |
| OLSZTYN | 65 | 26,912 | 34,353 | 453,882 | 67,689 | 707,256 |
| PIŁA | 121,5 | 5,892 | 3,367 | 310,759 | 17,022 | 358,897 |
| POZNAŃ | 382 | 3,530 | 3,231 | 387,923 | 56,828 | 499,796 |
| RADOM | 36,600 | 15,204 | 2,357 | 347,925 | 31,468 | 487,754 |
| SZCZECIN | 882 | 38,449 | 12,287 | 514,997 | 42,317 | 678,859 |
| SZCZECINEK | 473 | 58,656 | 20,839 | 441,927 | 25,748 | 610,829 |
| TORUŃ | 148 | 4,696 | 2,062 | 417,730 | 27,762 | 490,353 |
| WARSZAWA | 240 | 257 | 1,556 | 311,131 | 29,025 | 409,141 |
| WROCŁAW | 1,064 | 30,454 | 143,801 | 253,351 | 84,642 | 567,753 |
| ZIELONA GÓRA | 156 | 3,493 | 2,725 | 381,712 | 24,858 | 438,980 |
| ***Total*** | *269,678* | *549,650* | *451,786* | *6,140,837* | *710,746* | *9,242,439* |

**Model selection and averaging**

In the following, model selection and averaging tables are presented for each species, in alphabetic order. The third table in each species section represents the results of the temporal contributions analysis (Fernández-Martínez & Maspons 2019). See Materials and Methods for details.

**Global model of PST for all species (see Tables 1 and Methods and Materials for parameter definitions)**:

PST ~ age + PST1 + age:PST1 + T_wi_+ T_sp_ + T_su_ + T_au_ + P_wi_ + P_sp_ + P_su_ + P_au_ + SPEI + (1|site), family = “binomial”

***Abies alba***

**Table S2 Model selection results for drivers of PST in *Abies alba*.** Model selection results for global GLMM. Parameters (K), AIC, dAIC, and model weight. Only models with dAIC < 2 shown.

**Model K logLik AICc dAIC weight**

age + SPEI 4 -299.79 607.63 0.00 0.12

age + SPEI + T_sp_ + T_wi_ 6 -298.04 608.19 0.56 0.09

age + SPEI + PST1 + age:PST1 6 -298.07 608.23 0.60 0.09

age + SPEI + T_wi_ 5 -299.27 608.61 0.98 0.07

age + SPEI + T_sp_ 5 -299.51 609.08 1.45 0.06

age + SPEI + P_su_ 5 -299.51 609.10 1.46 0.06

age + SPEI + PST1 + T_sp_ + T_wi_ + age:PST1 8 -296.46 609.10 1.47 0.06

age 3 -301.56 609.14 1.51 0.06

age + SPEI + P_sp_ 5 -299.59 609.26 1.63 0.05

age + SPEI + PST1 + P_su_ + age:PST1 7 -297.57 609.27 1.64 0.05

age + SPEI + PST1 + T_sp_ + age:PST1 7 -297.58 609.30 1.66 0.05

age + SPEI + PST1 + T_sp_ + age:PST1 7 -297.69 609.52 1.88 0.05

age + SPEI + PST1 + T_wi_ + age:PST1 7 -297.70 609.55 1.91 0.05

age + SPEI + T_su_ 5 -299.76 609.59 1.95 0.05

age + SPEI + T_au_ 5 -299.76 609.59 1.96 0.05

age + SPEI + PST1 5 -299.76 609.60 1.97 0.05

**Table S3 Model averaging results for drivers of PST in *Abies alba.*** VI – variable importance, β – standardized parameter estimate, standard error, z-, and P-values for model-averaged parameters for all 15 models with dAIC < 2

**Variable VI β S.E. z *P .***

**age 1.00 0.588 0.117 5.0 < 0.001 *****

SPEI 0.94 -0.243 0.130 1.9 0.062 .

PST1 0.34 0.135 0.168 0.8 0.420

T_wi_ 0.32 0.193 0.151 1. 3 0.200

T_sp_  0.31 -0.212 0.174 1.2 0.223

age:PST1 0.29 -0.190 0.103 1.8 0.065 .

P_su_  0.16 0.128 0.142 0.9 0.368

T_su_  0.05 -0.049 0.186 0.3 0.792

T_au_  0.05 -0.031 0.121 0.3 0.798

P_sp_  0.05 0.072 0.114 0.6 0.527

**Table S4 Temporal contribution of significant predictors of PST1 to temporal trend in PST1 of *Abies alba***

**Parameter TC S.E. t P .**

**age 0.039 0.007 5.9 < 0.001** ***

***Fagus sylvatica***

**Table S5 Drivers of PST in *Fagus sylvatica*.** Model selection results for global GLMM. Parameters (K), AIC, dAIC, and model weight. Only models with dAIC < 2 shown.

**Model K logLik AICc dAIC weight**

age + PST1 + age:PST1 + SPEI 6 -311.31 634.73 0.00 0.17

age + PST1 + age:PST1 + SPEI + T_su_ 7 -310.57 635.27 0.55 0.13

age + PST1 + age:PST1 + SPEI + P_au_ 7 -310.71 635.56 0.83 0.11

age + PST1 + age:PST1 + SPEI + P_wi_ 7 -310.84 635.82 1.09 0.10

age + PST1 + age:PST1 + SPEI + T_wi_ 7 -311.01 636.16 1.43 0.08

age + PST1 + age:PST1 + SPEI + P_au_ + T_su_ 8 -310.01 636.19 1.47 0.08

age + PST1 + age:PST1 + SPEI + P_sp_ 7 -311.06 636.26 1.54 0.08

age + PST1 + age:PST1 + SPEI + T_sp_ 7 -311.12 636.38 1.66 0.07

age + PST1 + age:PST1 + SPEI + P_su_ 7 -311.14 636.42 1.69 0.07

age + PST1 + age:PST1 + SPEI + P_au_ + P_sp_ 8 -310.23 636.65 1.92 0.06

age + PST1 + age:PST1 + SPEI + T_au_ 7 -311.27 636.67 1.95 0.06

**Table S6 Model averaging results for drivers of PST in *Fagus sylvatica.*** VI – variable importance, β – standardized parameter estimate, standard error, z-, and P-values for model-averaged parameters for all 11 models with dAIC < 2

**Variable VI**^*^ **β S.E. z *P .***

**age 1.00 0.553 0.145 3.8** < 0.001 ***

**SPEI 1.00 -0.406 0.131 3.1** 0.002 **

PST1 1.00 -0.003 0.250 < 0.1 0.989

**age:PST1 1.00 -0.485 0.231 2.1** 0.035 *

P_au_ 0.25 0.118 0.104 1.1 0.257

T_su_ 0.20 -0.178 0.147 1.2 0.227

P_sp_ 0.14 -0.090 0.111 0.8 0.417

P_wi_ 0.10 0.101 0.104 1.0 0.330

T_wi_ 0.08 0.083 0.107 0.8 0.441

T_au_ 0.06 -0.033 0.105 0.3 0.764

T_sp_ 0.07 -0.074 0.120 0.6 0.539

P_su_ 0.07 0.075 0.127 0.6 0.556

**Table S7 Temporal contribution of significant predictors of PST1 to temporal trend in PST1 of *F. sylvatica***

**Parameter TC SE t P .**

**age 0.0052 0.0002 22.9 < 0.001 *****

**SPEI 0.0008 0.0002 4.4 < 0.001** ***

***Larix decidua***

**Table S8 Drivers of PST in *Larix decidua*.** Model selection results for global GLMM. Parameters (K), AIC, dAIC, and model weight. Only models with dAIC < 2 shown.

**Model K logLik AICc delta weight**

age 3 -344.27 694.57 0.00 0.14

age + PST1 4 -343.40 694.85 0.28 0.12

age + PST1 + age:PST1 5 -342.59 695.25 0.68 0.10

age + T_su_ 4 -343.91 695.88 1.31 0.07

age + T_au_ 4 -343.94 695.92 1.35 0.07

age + PST1 + T_su_ 5 -343.08 696.24 1.67 0.06

age + PST1 + T_au_ 5 -343.12 696.30 1.74 0.06

age + P_sp_ 4 -344.17 696.38 1.81 0.06

age + T_wi_ 4 -344.20 696.45 1.88 0.05

age + SPEI 4 -344.23 696.50 1.93 0.05

age + P_wi_ 4 -344.24 696.52 1.95 0.05

age + T_sp_ 4 -344.25 696.54 1.97 0.05

age + P_au_ 4 -344.25 696.56 1.99 0.05

age + P_su_ 4 -344.26 696.57 2.00 0.05

**Table S9 Model averaging results for drivers of PST in *Larix decidua.*** VI – variable importance, β – standardized parameter estimate, standard error, z-, and P-values for model-averaged parameters for all 14 models with dAIC < 2

**Variable VI**^*^ **β S.E. z *P .***

**age 1.00 0.163 0.073 2.2 0.026** *

PST1 0.34 13.130 25.300 0.5 0.604

T_su_ 0.13 -0.078 0.095 0.8 0.412

T_au_ 0.13 -0.070 0.088 0.8 0.431

age:PST1 0.10 -0.695 0.555 1.3 0.210

SPEI 0.05 0.029 0.101 0.3 0.771

T_sp_ 0.05 -0.016 0.075 0.2 0.833

P_su_ 0.05 0.018 0.122 0.1 0.882

P_au_ 0.05 -0.030 0.169 0.2 0.857

P_wi_ 0.05 0.060 0.230 0.3 0.795

P_sp_ 0.06 -0.095 0.211 0.5 0.653

T_wi_ 0.05 0.015 0.041 0.4 0.712

**Table S10 Temporal contribution of significant predictors of PST1 to temporal trend in PST1 of *L. decidua***

**Variable TC SE t P .**

**age 0.0026 0.0001 18.4 < 0.001** ***

***Picea abies***

**Table S11 Drivers of PST in *Picea abies*.** Model selection results for global GLMM. Parameters (K), AIC, dAIC, and model weight. Only models with dAIC < 2 shown.

**Model K logLik AICc dAIC weight**

age + PST1 + age:PST1 + SPEI + T_sp_ + T_wi_ 8 -426.99 870.13 0.00 0.12

age + PST1 + age:PST1 + T_sp_ + T_wi_ 7 -428.15 870.43 0.30 0.10

ate + PST1 + age:PST1 + SPEI + P_au_ + T_sp_ + T_wi_ 9 -426.53 871.26 1.13 0.07

age + PST1 + T_sp_ + T_wi_ 6 -429.60 871.28 1.15 0.07

age + PST1 + age:PST1 + SPEI + T_au_ + T_sp_ + T_wi_ 9 -426.67 871.53 1.40 0.06

age + PST1 + age:PST1 + SPEI + P_su_ + T_sp_ + T_wi_ 9 -426.70 871.59 1.46 0.06

age + PST1 + age:PST1 5 -430.85 871.76 1.63 0.05

age + PST1 + age:PST1 + SPEI + T_sp_ 7 -428.85 871.81 1.69 0.05

age + PST1 + age:PST1 + SPEI + P_wi_ + T_sp_ + T_wi_ 9 -426.81 871.81 1.69 0.05

age + PST1 + age:PST1 + SPEI + P_su_ + T_sp_ + T_wi_ 9 -426.81 871.82 1.69 0.05

age + PST1 + age:PST1 + T_sp_ 6 -429.87 871.84 1.71 0.05

age + PST1 + age:PST1 + P_wi_ + T_sp_ + T_wi_ 8 -427.88 871.91 1.78 0.05

age + PST1 + age:PST1 + P_au_ + T_sp_ + T_wi_ 8 -427.91 871.98 1.85 0.05

age + SPEI + T_au_ + T_sp_ + T_wi_ 7 -428.95 872.02 1.90 0.05

age + PST1 + age:PST1 + SPEI + P_su_ 7 -428.97 872.07 1.94 0.04

age + T_sp_ + T_wi_ 5 -431.00 872.07 1.94 0.04

age + PST1 + age:PST1 + SPEI + P_sp_ + T_sp_ + T_wi_ 9 -426.94 872.08 1.95 0.04

**Table S12 Model averaging results for drivers of PST in *Picea abies.*** VI – variable importance, β – standardized parameter estimate, standard error, z-, and P-values for model-averaged parameters for all 17 models with dAIC < 2

**Variable VI β S.E. z *P .***

**age                 1.00 0.416 0.102 4.1 < 0.001** ******

**T_sp_ 0.90 -0.255 0.124 2.0 0.041** *

PST1           0.84 0.195 0.102 1.9 0.053 .

**age:PST1      0.83 -0.194 0.092 2.1 0.034** *

T_wi_ 0.80 0.197 0.109 1.8 0.070 .

SPEI 0.66 -0.161 0.103 1.6 0.117

P_au_ 0.11 0.075 0.089 0.8 0.399

T_au_ 0.10 -0.087 0.093 0.9 0.352

T_su_ 0.10 -0.147 0.140 1.0 0.294

P_wi_ 0.05 0.061 0.092 0.7 0.506

P_su_ 0.05 0.062 0.105 0.6 0.554

P_sp_ 0.04 -0.027 0.089 0.3 0.767

**Table S13 Temporal contribution of significant predictors of PST1 to temporal trend in PST1 of *P. abies***

**Variable TC SE t P .**

PST1 0.0001 0.0002 0.5 0.308

**age 0.0044 0.0002 19.3 < 0.001** ***

T_sp_ < 0.0001 0.0003 < 0.1 0.493

***Pinus sylvestris***

**Table S14 Drivers of PST in *Fagus sylvatica*.** Model selection results for global GLMM. Parameters (K), AIC, dAIC, and model weight. Only models with dAIC < 2 shown.

**Model K logLik AICc dAIC weight**

age + PST1 + age:PST1 5 -514.25 1038.57 0.00 0.14

age + PST1 4 -515.27 1038.59 0.02 0.14

age + PST1 + age:PST1 + T_sp_ 6 -513.82 1039.73 1.16 0.08

age + PST1 + T_sp_ 5 -514.91 1039.88 1.31 0.07

age + PST1 + T_au_ 5 -514.97 1040.01 1.45 0.07

age + PST1 + age:PST1 + T_au_ 6 -513.97 1040.04 1.47 0.07

age + PST1 + P_su_ 5 -515.09 1040.26 1.69 0.06

age + PST1 + P_wi_ 5 -515.14 1040.35 1.78 0.06

age + PST1 + age:PST1 + P_su_ 6 -514.14 1040.37 1.80 0.06

age + PST1 + age:PST1 + P_wi_ 6 -514.15 1040.39 1.83 0.06

age + PST1 + age:PST1 + T_su_ 6 -514.21 1040.51 1.95 0.05

age + PST1 + age:PST1 + SPEI 6 -514.21 1040.52 1.95 0.05

age + PST1 + SPEI 5 -515.23 1040.53 1.96 0.05

age + PST1 + T_wi_ 5 -515.24 1040.54 1.97 0.05

**Table S15 Model averaging results for drivers of PST in *Pinus sylvestris.*** VI – variable importance, β – standardized parameter estimate, standard error, z-, and P-values for model-averaged parameters for all 14 models with dAIC < 2

**Variable VI**^*^ **β S.E. z *P .***

**age                1.00 0.239 0.089 2.7** 0.007 **

**PST1 1.00 0.245 0.088 2.8** 0.005 **

age:PST1 0.50 -0.116 0.081 1.4 0.154

T_sp_ 0.15 -0.075 0.084 0.9 0.371

T_au_  0.13 -0.058 0.077 0.8 0.448

P_su_  0.12 -0.042 0.078 0.5 0.593

P_wi_ 0.11 -0.037 0.077 0.5 0.631

SPEI         0.10 -0.022 0.080 0.3 0.777

T_wi_  0.05 0.021 0.078 0.3 0.784

T_su_  0.05 0.024 0.085 0.3 0.777

**Table S16 Temporal contribution of significant predictors of PST1 to temporal trend in PST1 of *P. sylvestris***

**Variable TC SE t P .**

**age 0.0028 0.0002 14.2 < 0.001 *****

**PST1 0.0007 0.0002 4.3 < 0.001 *****

***Quercus petraea***

**Table S17 Drivers of PST in *Quercus petraea*.** Model selection results for global GLMM. Parameters (K), AIC, dAIC, and model weight. Only models with dAIC < 2 shown.

**Model K logLik AICc dAIC weight**

age + PST1 + SPEI 5 -462.06 934.19 0.00 0.15

age + PST1 + SPEI + P_su_ 6 -461.53 935.14 0.95 0.09

age + PST1 + SPEI + P_sp_ 6 -461.55 935.18 0.99 0.09

age + SPEI 4 -463.58 935.20 1.01 0.09

age + SPEI + P_su_ 5 -462.83 935.72 1.53 0.07

age + PST1 + SPEI + T_wi_ 6 -461.85 935.79 1.60 0.07

age + PST1 + SPEI + Pau 6 -461.88 935.85 1.65 0.07

age + PST1 + SPEI + T_su_ 6 -461.89 935.88 1.69 0.07

age + SPEI + P_sp_ 5 -462.91 935.89 1.70 0.07

age + PST1 + SPEI + T_sp_ 6 -461.96 936.02 1.83 0.06

age + PST1 + SPEI + P_sp_ + P_su_ 7 -460.96 936.04 1.84 0.06

age + PST1 + SPEI + T_su_ 6 -462.04 936.16 1.97 0.06

age + PST1 + SPEI + age:PST1 6 -462.04 936.18 1.98 0.06

**Table S18 Model averaging results for drivers of PST in *Quercus petraea.*** VI – variable importance, β – standardized parameter estimate, standard error, z-, and P-values for model-averaged parameters for all models with dAIC < 2

**Variable VI**^*^ **β S.E. z *P .***

**age                1.00 0.273 0.097 2.8 0.005** **

**SPEI         1.00 -0.278 0.095 2.9 0.003** **

PST1         0.77 -0.139 0.087 1.6 0.110

P_su_  0.23 0.108 0.097 1.1 0.268

P_sp_  0.22 -0.092 0.087 1.1 0.289

T_wi_  0.09 0.056 0.086 0.7 0.513

P_au_  0.09 -0.052 0.086 0.6 0.544

T_su_  0.07 -0.069 0.119 0.6 0.560

T_sp_ 0.06 0.044 0.100 0.4 0.656

age:PST1     0.06 -0.022 0.106 0.2 0.837

P_wi_  0.06 -0.019 0.083 0.2 0.817

**Table S19 Temporal contribution of significant predictors of PST1 to temporal trend in PST1 of *Quercus petraea***

**Variable TC SE t P .**

**age 0.033 0.011 3.08 0.001** **

SPEI -0.007 0.008 -0.96 0.169

***Quercus robur***

**Table S20 Drivers of PST in *Quercus robur*.** Model selection results for global GLMM. Parameters (K), AIC, dAIC, and model weight. Only models with dAIC < 2 shown.

**Model K logLik AICc dAIC weight**

age + PST1 + SPEI 5 -466.27 942.61 0.00 0.17

age + PST1 + SPEI + P_su_ 6 -465.59 943.27 0.66 0.12

age + PST1 + SPEI + P_sp_ 6 -465.80 943.69 1.08 0.10

age + SPEI 4 -467.94 943.92 1.31 0.09

age + PST1 + SPEI + T_su_ 6 -465.99 944.07 1.46 0.08

age + SPEI + P_su_ 5 -467.03 944.13 1.52 0.08

age + PST1 + SPEI + P_sp_ + P_su_ 7 -465.06 944.25 1.63 0.08

age + PST1 + SPEI + T_sp_ 6 -466.11 944.30 1.69 0.07

age + PST1 + SPEI + T_su_ 6 -466.11 944.31 1.70 0.07

age + PST1 + SPEI + Pau 6 -466.14 944.38 1.77 0.07

age + PST1 + SPEI + age: PST1 6 -466.23 944.55 1.94 0.06

**Table S21 Model averaging results for drivers of PST in *Quercus robur.*** VI – variable importance, β – standardized parameter estimate, standard error, z-, and P-values for model-averaged parameters for all models with dAIC < 2

**Variable VI**^*^ **β S.E. z *P .***

**age                 1.00 0.296 0.098 3.0** 0.002 **

**SPEI 1.00 -0.293 0.097 3.0** 0.002 **

PST1          0.83 -0.148 0.088 1.7 0.093 .

P_su_ 0.28 0.119 0.097 1.2 0.218

P_sp_ 0.17 -0.085 0.086 1.0 0.324

T_wi_ 0.08 0.064 0.085 0.8 0.453

T_sp_ 0.07 0.057 0.099 0.6 0.563

T_su_ 0.07 -0.067 0.117 0.6 0.566

Pau 0.07 -0.043 0.085 0.5 0.612

age:PST1 0.06 -0.032 0.108 0.3 0.768

**Table S22 Temporal contribution of significant predictors of PST1 to temporal trend in PST1 of *Quercus robur***

**Variable TC SE t P .**

**age 0.041 0.009 4.72 < 0.001** ***

**SPEI -0.020 0.006 3.30 < 0.001** ***

**Table S23 Among-site synchrony (R ± S.E.) and temporal trends of weather parameters**. Synchrony was calculated in 10-year sliding windows and temporal trends determined by regressing the estimate against year.

| **Variable** |  | **R** | **SE** | ***P*** |  | **Trend** | **SE** | ***P*** |
| --- | --- | --- | --- | --- | --- | --- | --- | --- |
| Winter temperature | T_wi_ | **0.807** | **0.008** | < 0.001 |  | **0.002** | **0.000** | < 0.001 |
| Spring temperature | T_sp_ | **0.956** | **0.002** | < 0.001 |  | **0.002** | **0.000** | 0.001 |
| Summer temperature | T_su_ | **0.547** | **0.012** | < 0.001 |  | 0.001 | 0.001 | 0.357 |
| Fall temperature | T_au_ | **0.925** | **0.004** | < 0.001 |  | 0.000 | 0.001 | 0.690 |
| Winter precipitation | P_wi_ | **0.277** | **0.020** | < 0.001 |  | 0.000 | 0.001 | 0.980 |
| Spring precipitation | P_sp_ | **0.910** | **0.004** | < 0.001 |  | **-0.007** | **0.001** | < 0.001 |
| Summer precipitation | P_su_ | **0.419** | **0.013** | < 0.001 |  | 0.001 | 0.001 | 0.655 |
| Fall precipitation | P_au_ | **0.956** | **0.002** | < 0.001 |  | -0.003 | 0.001 | 0.073 |
| Drought index | SPEI | **0.876** | **0.006** | < 0.001 |  | 0.000 | 0.001 | 0.529 |
